# Green Tea Potentially Ameliorates Bisphenol A-Induced Oxidative Stress: An *In Vitro* and *In Silico* Study

**DOI:** 10.1155/2014/259763

**Published:** 2014-08-10

**Authors:** Hiral Suthar, R. J. Verma, Saumya Patel, Y. T. Jasrai

**Affiliations:** ^1^Department of Zoology, University School of Sciences, Gujarat University, Ahmedabad 380009, India; ^2^Department of Bioinformatics, Applied Botany Center, University School of Sciences, Gujarat University, Ahmedabad 380009, India

## Abstract

The present investigation was an attempt to elucidate oxidative stress induced by bisphenol A on erythrocytes and its amelioration by green tea extract. For this, venous blood samples from healthy human adults were collected in EDTA vials and used for preparation of erythrocytes suspension. When erythrocyte suspensions were treated with different concentrations of BPA/H_2_O_2_, a dose-dependent increase in hemolysis occurred. Similarly, when erythrocytes suspensions were treated with either different concentrations of H_2_O_2_ (0.05–0.25 mM) along with BPA (50 *μ*g/mL) or 0.05 mM H_2_O_2_ along with different concentrations of BPA (50–250 *μ*g/mL), dose-dependent significant increase in hemolysis occurred. The effect of BPA and H_2_O_2_ was found to be additive. For the confirmation, binding capacity of bisphenol A with erythrocyte proteins (hemoglobin, catalase, and glutathione peroxidase) was inspected using molecular docking tool, which showed presence of various hydrogen bonds of BPA with the proteins. The present data clearly indicates that BPA causes oxidative stress in a similar way as H_2_O_2_ . Concurrent addition of different concentrations (10–50 *μ*g/mL) of green tea extract to reaction mixture containing high dose of bisphenol A (250 *μ*g/mL) caused concentration-dependent amelioration in bisphenol A-induced hemolysis. The effect was significant (*P* < 0.05). It is concluded that BPA-induced oxidative stress could be significantly mitigated by green tea extract.

## 1. Introduction

Bisphenol A (BPA 2,2-bis(4-hydroxyphenyl) propane), a xenoestrogen, is an important monomer of polycarbonate plastics and a constituent of epoxy and polystyrene resins [1]. It is widely used to line metal cans, water pipes [2], baby bottles, drinking cups [3], dental sealants [4, 5], and many other household appliances. Studies have shown that for incomplete polymerization and for degradation of the polymer, bisphenol A can leach out from food and beverage containers [6], as well as from dental sealants and composites under normal conditions of use. Bisphenol A has been found not only in environmental samples, including air, water, sewage sludge, soil, and dust [7–9], but also in specimens of human body fluids, such as plasma, umbilical cord blood, placental tissue, amniotic fluid, follicular fluid, and breast milk. Due to the widespread use and building toxicological database, a need arises to investigate mechanism of bisphenol A-induced toxicity.

Studies revealed that bisphenol A causes adverse effects on brain, reproductive system, and liver [10, 11]. Mathur and D′Cruz [12] have reported that BPA causes an imbalance in the prooxidant and antioxidant status of the testis and increased amount of reactive oxygen species. A study carried out by Verma and Sangai [13] showed that treatment with bisphenol A causes cytotoxicity in human erythrocytes which may be due to the oxidative stress. But none of the studies have stated the exact mechanism of bisphenol A-induced oxidative damage.

We propose that bisphenol A causes generation of hydroxyl radical causing peroxidation of lipids. Erythrocytes have been used as a cellular model to investigate oxidative damage in biomembranes due to the presence of both high concentration of polyunsaturated fatty acids and the oxygen transport associated with redox active hemoglobin molecules, which are potent promoters of reactive oxygen species formation [14]. Along with that, H_2_O_2_, a potent endogenous oxidant of erythrocytes producing deleterious oxidative changes in the membrane by well-studied pathway, was chosen as standard compound to investigate/compare mechanism of bisphenol A activity.

Herbal medicines derived from plants are being increasingly utilized to treat wide variety of clinical diseases. More attention has been paid to the protective effects of natural antioxidants against drug-induced toxicities especially whenever free radical generation is involved [15]. Green tea demonstrated stronger free radical scavenging activity comparatively at lower doses* in vitro* [16]. Green tea leaves possess many beneficial properties to human health owning to the presence of many bioactive components, particularly the catechins that have intrinsic property of scavenging the reactive oxygen species produced in our body by various systems [17]. Green tea extract is known to have a broad spectrum of biological activities such as antioxidant, antiviral [18], anticancer [19], antibacterial [20], antifungal [21], and neuroprotective effects [22].

In the present study, we elucidate experimentally as well as by docking that bisphenol A induces oxidative stress similarly as H_2_O_2_ and green tea ameliorates changes caused by bisphenol A. Virtual screening of bisphenol A was targeted against hemoglobin, catalase, and glutathione peroxidase of erythrocytes via molecular docking studies and the docked conformations were evaluated in terms of binding efficiency. Xenobiotics-protein interaction studies have significance in obtaining a better insight in the mechanisms of toxicities. Thus, the molecular docking predictions were used to find the probable interacting sites of bisphenol A with hemoglobin, catalase, and glutathione peroxidase of erythrocytes.

## 2. Materials and Methods 

### 2.1. Extract Preparation

The green tea extract was prepared according to the method of Bhargava and Singh [23]. Briefly, 5 g of green tea powder (Brook-bond Company, Darjeeling green tea, India) was mixed with 100 mL 50% ethanol in water for 12 h with intermittent shaking every 2 h. The mixture was filtered with Whatman number 42 filter paper. The pooled filtrate was concentrated by evaporating below 50°C to obtain residue which was stored under refrigerated conditions. Residue was resuspended in normal saline before use.

### 2.2. Experimental Sample

Volunteers (healthy adult well-nourished human beings of 23–25 years of age) were recruited to participate in the study after approval of the Institutional Ethics Committee of the Zoology Department of Gujarat University, Ahmedabad. The venous whole blood samples were collected in syringes containing EDTA and centrifuged at 1,000 ×g for 10 min at 4°C to remove plasma and buffy coat. The RBCs were washed twice with cold saline (0.9% NaCl). The pellets were diluted with normal saline to obtain cell density of 2 × 10^4^ RBC/mL [24].

### 2.3. *In Vitro* Incubation of Erythrocytes with BPA, H_2_O_2_, and Green Tea Extract

The effects of BPA (Hi-media Laboratories Pvt. Ltd., Mumbai, India) and H_2_O_2_ (Merck Specialties Pvt. Ltd., Mumbai, India) on erythrocytes were investigated by incubating 2 mL of erythrocyte suspensions with (i) different concentrations of BPA (0–250 *μ*g/mL) and (ii) different concentrations of H_2_O_2_ (0–0.25 mM). Further, to investigate any interaction between BPA and H_2_O_2_, 2 mL of erythrocyte suspensions was incubated with (iii) 50 *μ*g/mL of BPA along with different concentrations of H_2_O_2_ (0–0.25 mM) and (iv) 0.05 mM of H_2_O_2_ along with different concentrations of BPA (0–250 *μ*g/mL). To study ameliorative effect of green tea extract against BPA, 2 mL of erythrocyte suspensions was incubated with different concentrations of green tea extract (0–50 *μ*g/mL) along with 250 *μ*g/mL concentration of BPA.

Total volume of each reaction mixture was made up to 4 mL with additional saline. All reaction mixtures were incubated at 37°C for 4 h with intermittent shaking.

### 2.4. Measurement of Hemolysis

The suspensions were centrifuged at 1,000 ×g for 10 min. The color density of the supernatant was read spectrophotometrically at 540 nm. Percent hemolysis was calculated by using the following formula:

(1) $$\begin{array}{c} \%\;\text{Hemolysis}=\frac{\text{Absorbance}\;\text{of}\;\text{individual}\;\text{tubes}}{\text{Absorbance}\;\text{with}\;100\%\;\text{hemolysis}}\times 100. \end{array}$$

To obtain 100% hemolysis, 2 mL of erythrocytes suspension was incubated with 2 mL of distilled water.

### 2.5. Statistical Analysis

Each experiment was repeated ten times. The results were expressed as the means ± SEM. The data were statistically analyzed using one-way analysis of variance (ANOVA) followed by Tukey's post hoc test. The level of significance was accepted with *P* < 0.05. Correlation coefficient was measured to estimate the strength of linear association between two variables.

### 2.6. Preparation of Protein Target Structure and Ligands

The X-ray crystal structure of hemoglobin (PDB id-2HHB), catalase (PDB id-1QQW), and glutathione peroxidase (PDB id-2f8A) was retrieved from the Protein Data Bank (PDB ID-2YK0) [25]. The Data was subjected to energy minimization using GROMOS96 utility (without reaction field) implemented in Swiss-Pdb Viewer 4.0.1. [26].

Ligand structure of bisphenol A (2D structure) was retrieved in structure data format (SDF) from NCBI PubChem [27] and 3D structure was derived using ChemSketch v. 10 [28]. The structure of bisphenol A was energy minimized using molecular mechanics geometry optimization module implemented in HyperChem [29]. AMBER force field with distant dependent dielectric constant, scale factor for electrostatic and van der Waals forces set to 0.5 and without any cutoffs to bond types, and its lengths were chosen to determine global minimum energy conformation. Subsequently, all the structures were minimized and exported to hard disk.

### 2.7. Active Site Prediction

This prediction was not carried out for protein structures cocrystallized with ligand as the ligand binding site was implicated as active site for docking with the ligand dataset. Structures with unbound ligands were computationally analyzed for active site using Q-SiteFinder [30]. It detects pockets on the protein surface through calculation of van der Waals interaction energies using a methyl probe and probes with favorable interaction energies were clustered and ranked.

### 2.8. Virtual Screening

Bisphenol A under study was virtually screened (docked) into the binding site of the target proteins as hemoglobin (PDB id-2HHB), catalase (PDB id-1QQW), and glutathione peroxidase (PDB id-2f8A) using ArgusLab 4.0.1 from Planaria Software LLC [31]. To enable fast sampling, the binding site was constructed which consists of all residues that have at least one atom within 3.5 Å from any atom in the cocrystallized ligand. This procedure of constructing binding site was applied for protein structures with cocrystallized ligand. A different approach was performed for proteins with unbound ligand whose active site was predicted using Q-SiteFinder. Best 3 scored pockets were computationally analyzed for each protein target using Jmol Java plugin implemented in Q-SiteFinder and the amino acids embedded in the predicted cavity volume were utilized as active site residues.

These two approaches generally gave a good representation of the important residues in the binding pocket for a protein target. A grid box of size X = 22 × Y = 15 × Z = 16 with atom scaling of 0.40 Å was generated and high precision ArgusDock engine with a score as scoring function was selected. After grid generation, the ligands were flexibly docked with the protein and 1000 poses were generated, among which best 10 poses of low energy which were clustered in rank 1 were examined. ArgusDock engine makes use of ligand torsionality as a hierarchical tree in which the root's node (group of bonded atoms that do not have rotatable bonds) is placed in a search point inside a grid comprised of residues of the active site. A set of diverse and energetically favorable translations are generated and poses that survive in torsional search through an approximate exhaustive search are retained and finally clustered.

## 3. Results

### 3.1. Effect on Erythrocytes

When erythrocyte suspensions were incubated with normal saline, ambient supernatant was clear with intact erythrocytes pellet in the bottom of the tube. Almost no hemolysis was observed in this mixture.

**Figure 1:**
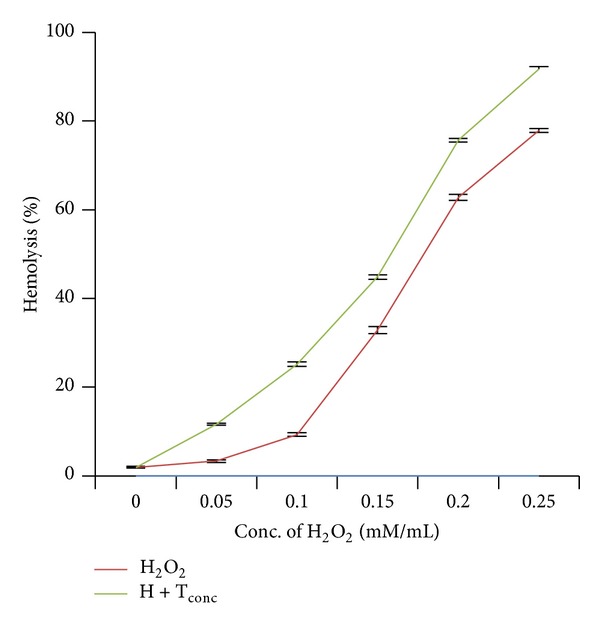
Effect of hydrogen peroxide and its combination with bisphenol A on red blood corpuscles. Results are expressed as mean ± SEM; *n* = 10. ∗Significant at the level *P* < 0.05 as compared to control.

Addition of various concentrations of H_2_O_2_ (0.05–0.25 mM) to erythrocyte suspensions caused significant (*P* < 0.05) increase in hemolysis (Figure 1). Maximum hemolysis (77.88%) was obtained on addition of 0.25 mM of H_2_O_2_ (Table 1). The results showed that H_2_O_2_ induced hemolysis in a concentration-dependent manner (*r* = 0.95).

**Figure 2:**
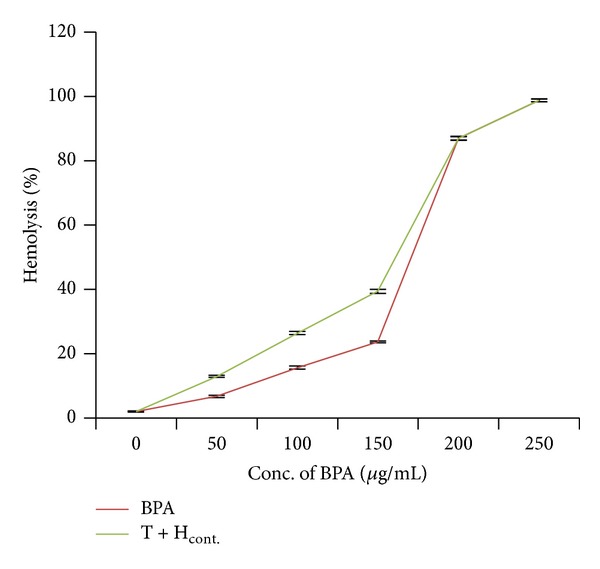
Effect of bisphenol A alone and its combination with hydrogen peroxide on red blood corpuscles. Results are expressed as mean ± SEM; *n* = 10. ∗Significant at the level *P* < 0.05 as compared to control.

Addition of different concentrations (50–250 *μ*g/mL) of bisphenol A to erythrocyte suspensions resulted in significant (*P* < 0.05) increase in hemolysis (Figure 2). With increasing concentrations of bisphenol A, amount of intact erythrocytes settled down at the bottom of the tubes got reduced drastically resulting in reddish supernatant. Maximum hemolysis (98.75%) was achieved on addition of 250 *μ*g/mL of bisphenol A (Table 2). The result showed that hemolysis induced by bisphenol A is dose-dependent (*r* = 0.91).

In another set of experiments, we studied effect of cotreatments of H_2_O_2_ and bisphenol A on hemolysis. When erythrocyte suspensions were incubated with different concentrations of H_2_O_2_ (0.05–0.2 mM) along with the 50 *μ*g/mL of bisphenol A, it caused significant (*P* < 0.05) increase in hemolysis (Figure 1).  Table 3 showed that when erythrocytes were treated with 0.05, 0.10, and 0.15 mM/mL of H_2_O_2_, hemolysis achieved was 3.36%, 9.33%, and 32.85%, which reached 12.93%, 25.18%, and 44.82% upon addition of 50 *μ*g/mL of BPA, respectively. The effect was also dose-dependent (*r* = 0.98).

In the same manner, when erythrocyte suspensions were cotreated with 0.05 mM of H_2_O_2_ along with different concentrations (50–250 *μ*g/mL) of bisphenol A, it caused significant (*P* < 0.05) increase in hemolysis (Figure 2).  Table 4 showed that when erythrocytes were treated with 50, 100, and 150 *μ*g/mL of bisphenol A, hemolysis achieved was 6.71%, 15.60%, and 23.59%, which reached 12.93%, 26.37%, and 39.37% upon addition of H_2_O_2_ (0.05 mM), respectively. The effect was dose-dependent (*r* = 0.97). Tables 3 and 4 showed that this acceleration of hemolysis could be due to the additive effect of both the compounds.

**Figure 3:**
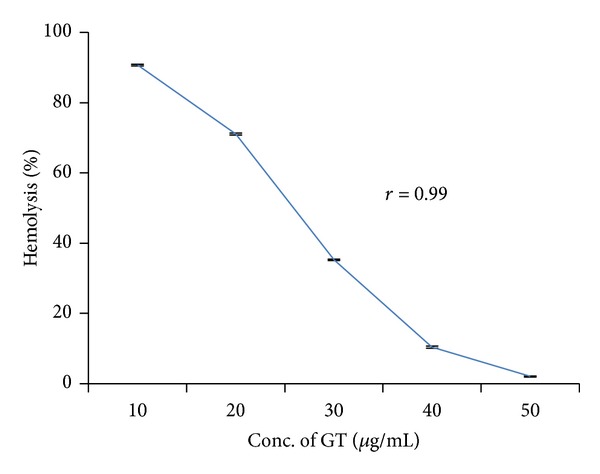
Retardation in bisphenol A-induced hemolysis by green tea extract. Results are expressed as mean ± SEM; *n* = 10. ∗Significant at the level *P* < 0.05 as compared to control.

Addition of only green tea extract into erythrocyte suspensions resulted in hemolysis (1.67%) almost equal to control tubes treated with normal saline. When erythrocytes were incubated with different concentrations of green tea extract (10–50 *μ*g/mL) along with 250 *μ*g/mL of bisphenol A, it caused significant (*P* < 0.05) and concentration-dependent reduction in hemolysis (*r* = 0.99) (Figure 3). This protective effect was highest at 50 *μ*g/mL concentration of green tea extract.

### 3.2. Computational Docking and Modeling Studies

We implemented a structure-based computational approach to investigate the potential interaction pattern of bisphenol A against hemoglobin, catalase, and glutathione peroxidase. The ligand dataset was virtually screened with the protein targets using ArgusLab software and the binding energy values were analyzed for each docked conformation. To identify the precise binding sites on hemoglobin, catalase, and glutathione peroxidase, molecular docking simulations were done with the binding mode of bisphenol A. Bisphenol A was docked into the X-ray crystal structures of hemoglobin (PDB id-2HHB), catalase (PDB id-1QQW), and glutathione peroxidase (PDB id-2f8A).

**Figure 4:**
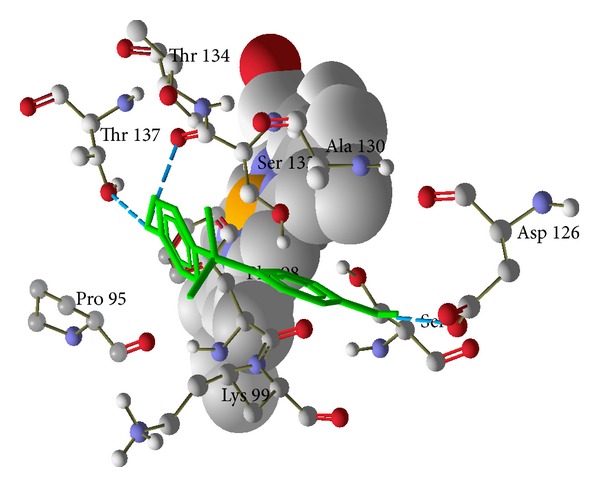
Hydrogen bond and steric interactions of human deoxyhaemoglobin (PDBid-2HHB) with bisphenol A. Green color is bisphenol A and whole structure is hemoglobin.

**Figure 5:**
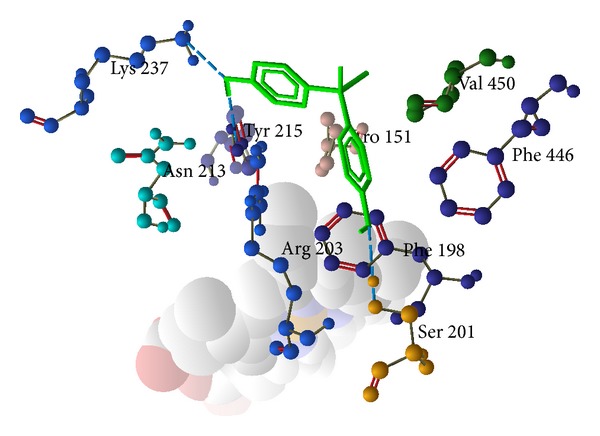
Hydrogen bond and steric interaction of human catalase (PDB id-1QQW) with bisphenol A. Green color is bisphenol A and whole structure is of catalase.

**Figure 6:**
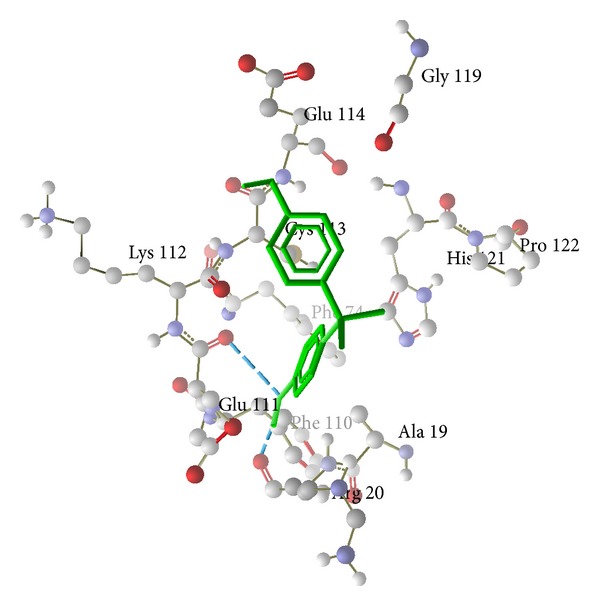
Hydrogen bond and steric interaction of human glutathione peroxidase (PDB id-2F8A) with bisphenol A. Green color is bisphenol A and whole structure is glutathione peroxidase.

The results showed that bisphenol A has higher binding affinity with the cavity present in hemoglobin and possessed greater energy (−79.5491 kcal/mol) value than the others, namely, catalase (−36.9179 kcal/mol) and glutathione peroxidase (−71.8106 kcal/mol) (Table 5). Conformations having low energy and exhibiting favorable hydrogen bonding with the amino acids side chain and its amide nitrogen were considered. The docking stimulations revealed the most likely binding site within the cavity of enzyme. Table 5 shows the amino acid residues interacting with the binding site of hemoglobin were Lys 99, Thr 137, Ser 133, and Asp126 (H-bond and steric interaction—Figure 4), binding sites of catalase were Lys 237, Tyr 215, Arg 203, Phe 198, Ser 201, and Phe 446 (H-bond and stearic interactions—Figure 5), and binding sites of glutathione peroxidase were Arg 20, Phe 110, Glu 111, His 121, and Glu 114 (H-bond and stearic interactions—Figure 6). The essential driving force of this binding site was hydrogen bond.

## 4. Discussion 

Results of the present study revealed that treatment of H_2_O_2_ to the erythrocyte suspensions caused dose-dependent increase in hemolysis. Aslan et al. [32] have also reported increased osmotic fragility of erythrocyte suspensions treated with H_2_O_2_. Interaction of H_2_O_2_ with iron of heme results in the generation of more potent hydroxyl radicals [33, 34], causing increased lipid peroxidation by attacking membrane polyunsaturated fatty acids. This is a known mechanism of action of H_2_O_2_ causing oxidative stress.

Being lipophilic in nature [35], BPA may get stabilized in the hydrophobic plasma membrane of the cell and may initiate peroxide formation. It may cross the lipid membrane and bind to iron of hemoglobin resulting in dissociation of hemoglobin subunits and release of iron which itself acts as prooxidant and produces high amount of hydroxyl radicals. Generated hydroxyl radicals may disturb the lipid membrane and initiate lipid peroxidation making the cell osmotically more fragile and ultimately causing hemolysis.

Earlier, it has been shown that bisphenol A induced oxidative stress in different tissues in rat [36, 37] and induced cellular apoptosis in hepatocytes [38]. Moon et al. [39] indicated that bisphenol A can induce hepatic damage and mitochondrial dysfunction by increasing oxidative stress in the liver. Sajiki [40] confirmed that bisphenol A binds to ferric heme of red blood corpuscle and Obata and Kubota [41] reported that bisphenol A increased hydroxyl radical formation in the rat striatum in a study using* in vivo* microdialysis. Another study done by Verma and Sangai [13] reported significant and concentration-dependent rise in lipid peroxidation in liver and kidney homogenates treated with bisphenol A in* in vitro* experiments, which supports our study.

**Figure 7:**
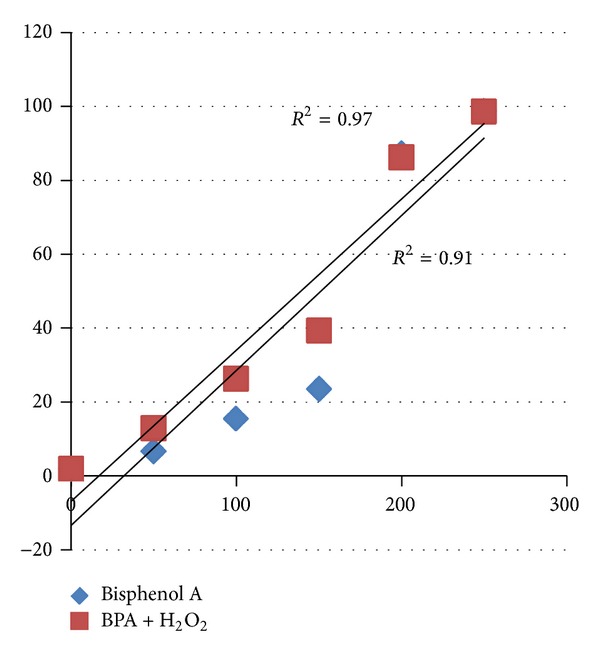
Correlation between bisphenol A and different concentration of bisphenol A along with the H_2_O_2_ (0.05 mM).

**Figure 8:**
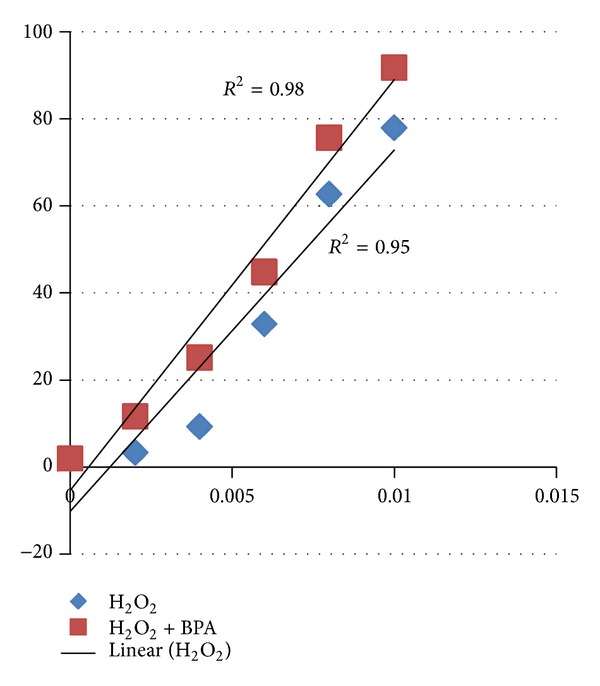
Correlation between H_2_O_2_ and different concentration of H_2_O_2_ along with the bisphenol A (50 *μ*g/mL).

The combinational studies (BPA and H_2_O_2_, Tables 3 and 4) have accelerated hemolysis which could be due to additive effect of both the compounds. The above hypothesis was clearly proven as the hemolysis induced by bisphenol A and H_2_O_2_ was highly correlated (Figures 7 and 8).

As shown in Figure 3, green tea extract significantly (*P* < 0.05) reduced hemolysis induced by bisphenol A. Another study of Verma and Sangai [13] has reported ameliorative effect of black tea extract (0–200 *μ*g/mL) on bisphenol A-induced cytotoxicity. Maximal retardation in hemolysis was observed with 200 *μ*g/mL concentration in case of black tea extract, which was 50 *μ*g/mL concentration in green tea extract. It clearly indicates that green tea extract is comparatively more potent than the black tea extract. Polyphenols found in green tea show 20 times more powerful antioxidant activity than vitamin C [42]. Several studies [43–45] have reported environmental contaminants-induced cytotoxic effects on erythrocytes and their amelioration by natural antioxidants.

Green tea extract contains catechins, well known for their antioxidative property, stabilized plasma membrane of erythrocytes, and reducing hemolysis [46]. Catechins can also chelate metal ions such as iron (III) to form inactive complexes and prevent the generation of potentially damaging free radicals [46, 47]. Green tea extract is considered as potent scavengers of reactive oxygen species, such as superoxide, hydrogen peroxide, hydroxyl radicals, and nitric oxide produced by various chemicals [48]. In recent study, supplementation of green tea extract attenuates cyclosporine A-induced oxidative stress in rats [49]. Ostrowska et al. [50] revealed that green tea may protect liver and brain cells against oxidative stress induced by ethanol intoxication.

Mathur et al. [51] explained that bisphenol A elicits depletion of antioxidant defense system and induces oxidative stress. Another study done by Hassan et al. [52] reported that bisphenol A induces hepatotoxicity through oxidative stress and ultimately decreases the antioxidant enzymes. Catalase and glutathione peroxidase are important enzymes of antioxidant defense systems, which protect tissue against oxidative stress induced by reactive oxygen species. Both these enzymes catalyze the hydrolysis of H_2_O_2_ into water and oxygen molecules to prevent the tissue injury.

We propose that bisphenol A increases hemolysis by increasing the formation of hydroxyl radicals which may directly or indirectly (through the inhibition of glutathione peroxidase and catalase) induce lipid peroxidation. For confirmation of this hypothesis, we have performed molecular docking of bisphenol A with hemoglobin, catalase, and glutathione peroxidase. Molecular docking approach typically uses an energy-based scoring function to identify the energetically most favorable ligand conformation with the bound target. Binding energies of the protein-ligand interactions are important to describe how fit the ligand binds to the target macromolecule. The general hypothesis is that lower energy scores represent better protein-ligand bindings compared to higher energy values. The ligand-binding mode with the lowest energy docking simulations of bisphenol A against proteins (hemoglobin (PDB id-2HHB), catalase (PDB id-1QQW), and glutathione peroxidase (PDB id-2f8A)) were evaluated based on the binding compatibility (docked energy (kcal/mol)) with the receptor (Figures 4, 5, and 6). On the basis of the result, we conclude that the binding affinity of bisphenol A is higher with the hemoglobin (−79.5491 kcal/mol) compared to the catalase (−36.9179 kcal/mol) and glutathione peroxidase (−71.8106 kcal/mol) as shown in the result. The docking results revealed that no van der Waals, hydrophobic, or electrostatic interactions existed but only hydrogen bond played a major role in the binding of bisphenol A against hemoglobin, catalase, and glutathione peroxidase. We sought to determine and explore the mechanism of bisphenol A by binding more efficiently with heme than the catalase and glutathione peroxidase, produce hydroxyl radicals like H_2_O_2_, and induce lipid peroxidation.

In conclusion, we report that bisphenol A exerts cytotoxicity on human erythrocytes through the production of hydroxyl radical in the same manner as hydrogen peroxide. Green tea extracts have beneficial effects on bisphenol A-induced cytotoxicity due to their potent antioxidant properties and ability to reduce oxidative stress.

## Figures and Tables

**Table 1:** H_2_O_2_ induced hemolysis.

Concentration of H_2_O_2_ (mM)	Hemolysis (%)
0 (control)	1.97 ± 0.14
0.05	3.36 ± 0.28
0.10	9.33 ± 0.37∗
0.15	32.85 ± 0.76∗
0.20	62.74 ± 0.68∗
0.25	77.88 ± 0.44∗

Results are expressed as mean ± SEM; *n* = 10.

∗Significant at the level *P* < 0.05 as compared to control.

**Table 2:** Bisphenol A induced hemolysis.

Concentration of bisphenol A (*μ*g/mL)	Hemolysis (%)
0 (control)	1.97 ± 0.14
50	6.71 ± 0.33∗
100	15.60 ± 0.49∗
150	23.59 ± 0.30∗
200	86.97 ± 0.55∗
250	98.75 ± 0.47∗

Results are expressed as mean ± SEM; *n* = 10.

∗Significant at the level *P* < 0.05 as compared to control.

**Table 3:** H_2_O_2_ induced hemolysis along with cotreatment of bisphenol A.

Treatment		Hemolysis (%)
H_2_O_2_ concentration (mM)	Bisphenol A concentration (*µ*g/mL)	
0 (control)	0 (control)	1.97 ± 0.14
0	50	6.71 ± 0.33∗
0.05	50	12.93 ± 0.37∗
0.10	50	25.18 ± 0.50∗
0.15	50	44.81 ± 0.50∗
0.20	50	75.69 ± 0.31∗
0.25	50	91.73 ± 0.47∗

Results are expressed as mean ± SEM; *n* = 10.

∗Significant at the level *P* < 0.05 as compared to control.

**Table 4:** Bisphenol A induced hemolysis along with cotreatment of H_2_O_2_.

Treatment		Hemolysis (%)
Bisphenol A concentration (*μ*g/mL)	H_2_O_2_ concentration (mM)	
0 (control)	0 (control)	1.97 ± 0.14
0	0.05	3.36 ± 0.28
50	0.05	12.93 ± 0.37∗
100	0.05	26.37 ± 0.45∗
150	0.05	39.37 ± 0.58∗
200	0.05	86.89 ± 0.58∗
250	0.05	98.73 ± 0.42∗

Results are expressed as mean ± SEM; *n* = 10.

∗Significant at the level *P* < 0.05 as compared to control.

**Table 5:** Docking results of human erythrocyte (PDB id-2HHB), catalase (PDB id-1QQW), and glutathione peroxidase (PDB id-2F8A) with bisphenol A.

Name	Target molecules	Docking score	Interaction	H-bond	Amino acid interactions	
					H-bond interactions	Steric interaction
Bisphenol A	Hemoglobin	−79.5491	−99.0041	−4.80926	Thr 137, Ser 133, Asp 126	Lys 99, Thr 137
Bisphenol A	Catalase	−36.9179	−54.8291	−3.275	Lys 237, Tyr 215, Ser 201,	Tyr 215, Arg 203, Phe 198, Phe 446
Bisphenol A	Glutathione peroxidase	−71.8106	−86.9671	−3.44834	Arg 20, Glu 111, Glu 114	Phe 110, Glu 114, His 121,
